# Ovarian preservation improves overall survival in young patients with early-stage endometrial cancer

**DOI:** 10.18632/oncotarget.18404

**Published:** 2017-06-07

**Authors:** Peng Jia, Yan Zhang

**Affiliations:** ^1^ Department of Obstetrics and Gynecology, Peking University First Hospital, Beijing, People's Republic of China

**Keywords:** endometrial cancer, early-stage, ovarian preservation, young women, meta-analysis

## Abstract

We searched Medline, Embase, Cochrane library, the Chinese Biomedicine Literature Database, the Chinese Scientific Journal Full-text Database, the Chinese Journal Full-text Database, and the Wanfang Database to collect observational studies on the effects of ovary-saving surgery in comparison to bilateral salpingo-oophorectomy (BSO) in young patients with early-stage endometrial cancer (EC). The literature search included studies up to March 2017, and 10 retrospective cohort studies met our selection criteria. Random and fixed effect models revealed that ovarian preservation (OP) was associated with better overall survival (OS) (hazard ratio [HR] 0.75, 95% confidence interval [CI] 0.57–0.99, *P* = 0.044), and was not associated with reduced recurrence-free survival (RFS) in pre-menopausal patients with early-stage endometrial cancer (HR 1.22, 95% CI 0.32–4.72, *P* = 0.648; risk ratio [RR] 1.11, 95% CI 0.59–2.10, *P* = 0.745). Preservation of the ovaries appears to be a safe option with significant benefit for this low risk population after a thorough preoperative evaluation and extensive intraoperative exploration.

## INTRODUCTION

EC is primarily a disease of postmenopausal women, but 25% of patients are premenopausal [1]. The incidence of EC in women under the age of 40 years is reported to be 2–14% and has been increasing in recent years [2]. The prognosis for premenopausal women with early-stage EC is favorable, with a 5 year survival rate greater than 90% [3]. Younger women are more likely to be diagnosed with earlier stage disease than their older counterparts, and their overall mean survival is significantly better [4]. However, the standard surgical staging treatment, which has not been changed since 1988, consists of total abdominal hysterectomy and BSO with pelvic and paraaortic lymphadenectomy as needed, regardless of the age of the patient or the stage of tumor. Removing both ovaries in premenopausal women leads to symptoms of menopause, fertility loss, and increased risk of cardiovascular disease, that seriously reduce the quality of post-operative life [5]. Recent studies have found that the incidence of ovarian metastasis is only ~5% in patients with clinical early-stage EC and it can be negligible in the absence of intraoperative evidence of advanced disease [6, 7]. Some studies have evaluated the oncological prognosis of early-stage EC patients with ovarian preservation and found no significant differences with BSO [8, 9]. Since no prospective study on this issue has been designed, and few large sample retrospective studies have been performed, a systematic review and meta-analysis may carry weight. We analyzed the related literature and performed a meta-analysis in order to reveal whether BSO provided any added benefit for the survival of young women with early-stage EC.

## RESULTS

### Search results

**Figure 1:**
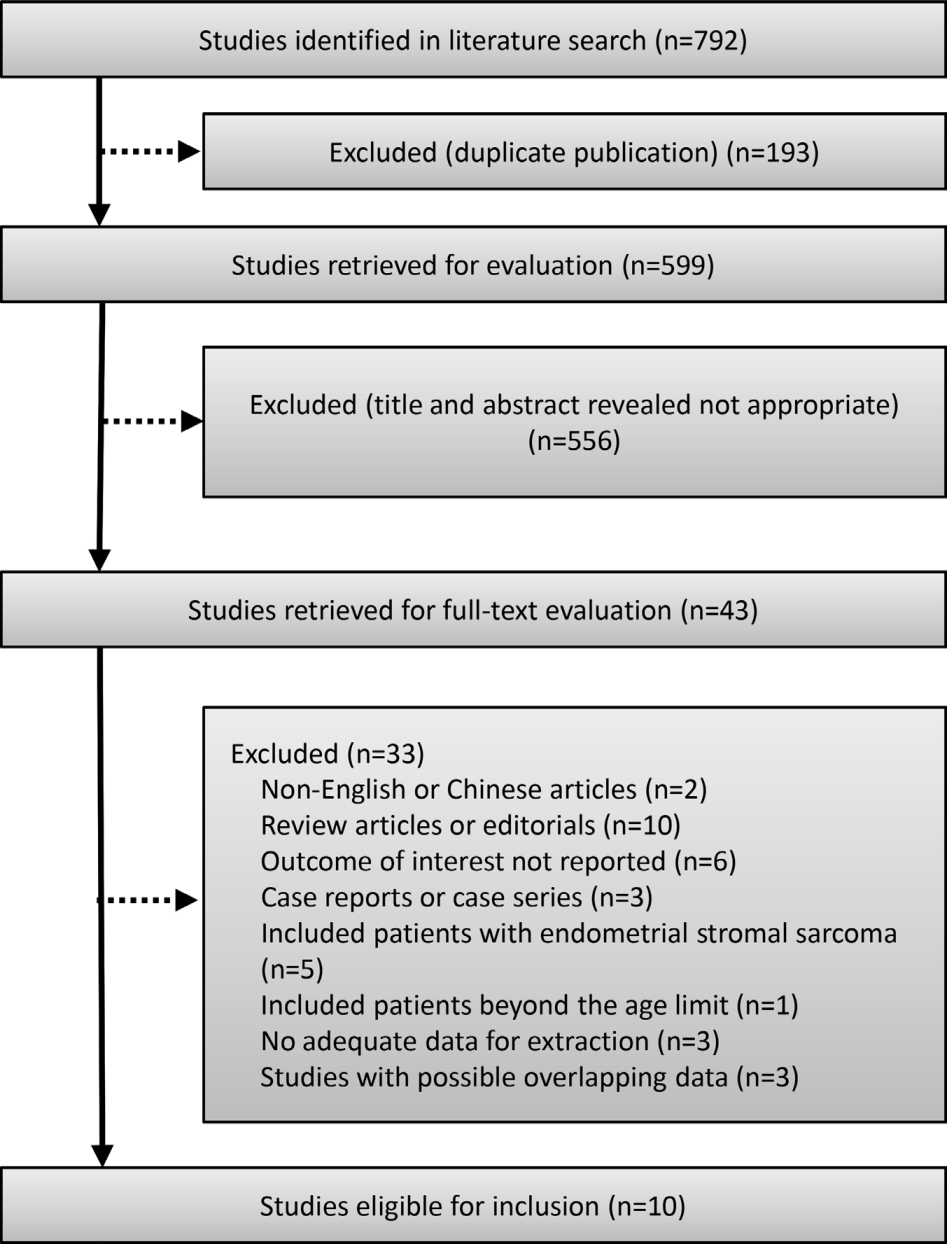
Flow chart of the study selection

The search strategy generated 792 citations, of which 43 were potentially relevant and retrieved for assessment (Figure 1). Of these, 33 were excluded for various reasons, leaving 10 retrospective cohort studies to perform our meta-analysis [10–19]. We dropped one study because it might comprise the same study population as two other studies from the USA, although the data was extracted from a different database [11, 19, 20]. We chose to keep the two studies that analyzed data from the Surveillance, Epidemiology, and End Results program (SEER) rather than the one from the National Cancer Database ( NCDB ) due to their longer follow-up duration [11, 19]. We dropped another study from Taiwan because it contained patients over the upper age limit [21]. Two studies were dropped from the meta-analysis because they did not present the measured HR, so that we could not extract the original data to calculate pooled HR [18, 22]. Although we could estimate the HRs from the Kaplan-Meier curves in these studies, these curve methods to estimate HR are likely to be the least reliable. Therefore, we did not use the data to perform the meta-analysis. All of the studies included were classified high quality (scored 6 stars or more) according to the Newcastle-Ottawa Scale (NOS) designed for retrospective cohort studies.

The clinical characteristics of all patients and methodological qualities from the 10 articles are summarized in Table 1. Data were extracted from studies conducted in the USA, Korea, and China. Tumors were restaged according to the International Federation of Gynecology and Obstetrics (FIGO) revised 2009 system for EC, and were classified as stage INOS (not otherwise specified) if the depth of myoinvasion was not available [23]. All of the patients selected were diagnosed with early-stage tumors (stages I–II), and most of them were stage Ia with a histological type of grade 1 endometrioid adenocarcinoma.

**Table 1:** Summary table of selected studies

Study	Year	Country	Data Source	Period	NO.(OP/BSO)	Age (y)	Stage	Grade	Type	Follow-up (m)	NOS
Gonthier [11]	2017	USA	SEER	1983–2012	96/849	≤ 45	Ia	G2 90%	Endo	0–352	9
								G3 10%			
Koji [19]	2016	USA	SEER	1983–2012	1034/8076	≤ 50	Ia 87%	G1	Endo	0–360	9
							Ib 3%				
							INOS 10%				
**Lee** [17]	2013	Korea	Koreal GOG	1997–2008	176/319	PRE	Ia 89%	G1 78%	Endo	6–208	8
							Ib 5%	G2 18%			
							II 6%	G3 4%			
**Sun** [15]	2013	China	Tongji Hospital	2000–2010	34/132	≤ 45	Ia 93%	G1 66%	Endo 97%	27–122	8
							Ib 7%	G2 21%			
								G3 13%			
Richter [18]	2009	USA	YNHH	1960–2006	20/153	≤ 45	I	N	N	0–480	7
**Li** [14]	2014	China	CHCAMS	1999–2012	20/55	≤ 40	Ia 69%	G1 71%	Endo	0.3–160	8
							Ib 31%	G2 25%			
								G3 4%			
Yang [12]	2016	China	SPTH	2008–2010	35/25	≤ 40	I	G1 82%	Endo	36	7
								G2 18%			
**Li** [16]	2013	China	PCH	1998–2008	17/31	≤ 45	Ia 71%	N	Endo 88%	120	6
							Ib 29%				
Wang [13]	2016	China	BOGH	2009–2015	25/76	≤ 45	Ia 87%	G1 75%	Endo 99%	3–72	7
							Ib 13%	G2 21%			
								G3 40%			
Wang [10]	2017	China	PUMCH	2005–2011	25/47	≤ 45	Ia 90%	G1 78%	Endo	7–131	8
							Ib 10%	G2 13%			
								G3 9%			

Note: BSO = bilateral salpingo-oophorectomy; OP = ovarian preservation; NOS = not otherwise specified; N = not mentioned; DFS = disease free survival; PRE = premenopausal; Endo = endometrioid adenocarcinoma; SEER = the Surveillance, Epidemiology, and End Results program; YNHH = Yeal-New Haven Hospital; CHCAMS = Cancer Hospital of Chinese Academy of Medical Sciences; SPTH = Shaanxi Provincial Tumor Hospital; PCH = Panyu Central Hospital; BOGH = Beijing Obstetrics and Gynecology Hospital; PUMCH = Peking Union Medical College Hospital.

### Ovarian preservation correlates with improved OS

**Figure 2:**
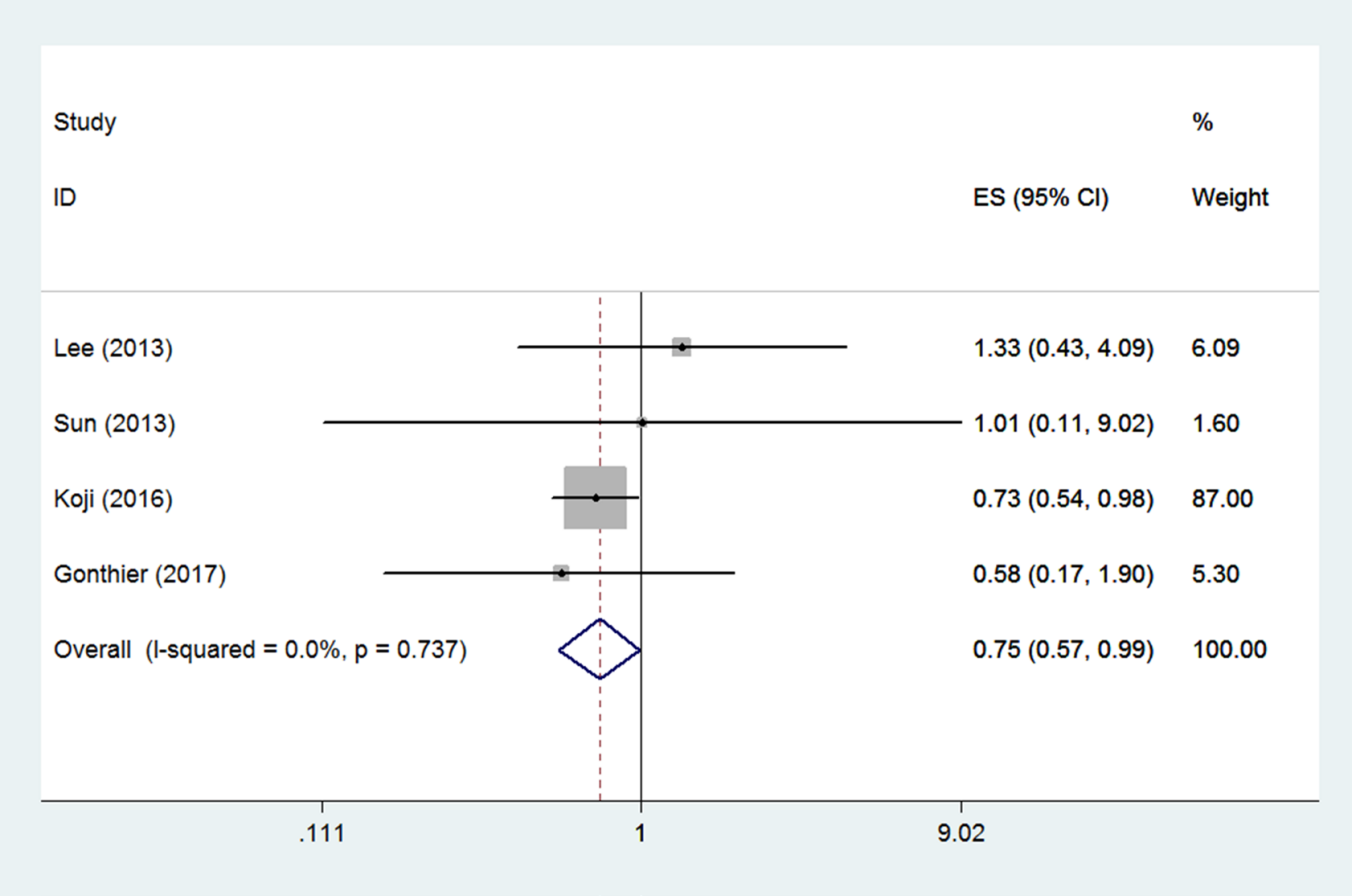
Forest plots illustrate the intervention results of the HRs for OS

Four studies from the USA, Korea, and China compared OS between the BSO group and the OP group in 10716 patients (9376/1340) expressed as HR [11, 15, 17, 19]. Fixed effect model analysis showed that OP was significantly associated with improved OS of young patients with early-stage EC (HR 0.75, 95% CI 0.57–0.99, *P* = 0.044 Figure 2), with no statistically significant heterogeneity detected between studies (*I*^2^ = 0.0%, *P* = 0.737) (Figure 5A) and no evidence of publication bias (Egger test, *P* = 0.558, Begg test, *P* = 0.734). Of the four studies, one from China contained a small number of patients with non-endometrioid histological type. When only the three studies that were restricted to patients with endometrioid adenocarcinoma were considered in the analysis the HR of OS was of a similar magnitude (HR 0.75, 95% CI 0.57–0.99, *P* = 0.043).

### No benefit of BSO over OP in RFS

**Figure 3:**
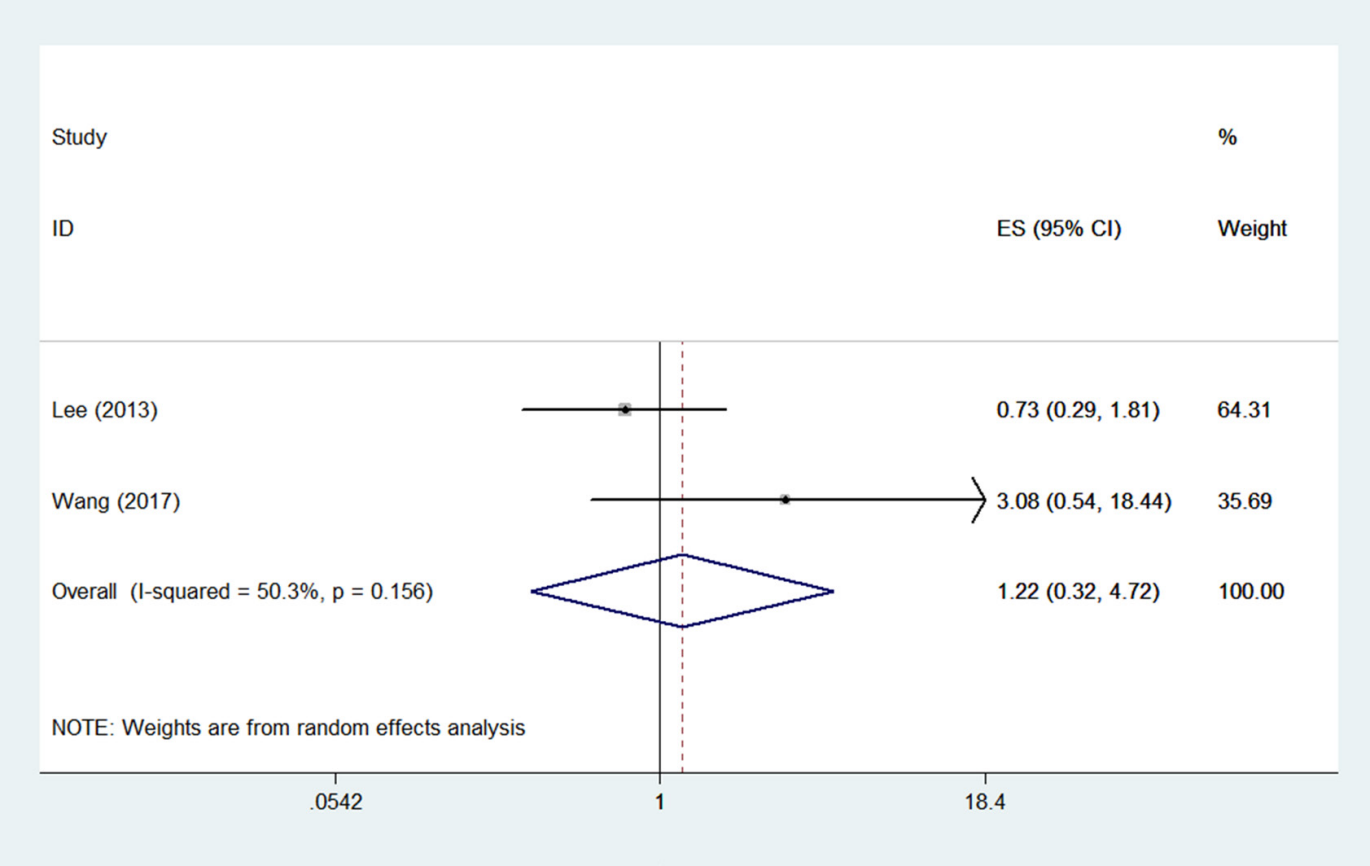
Forest plots illustrate the intervention results of the HRs for RFS

Two studies compared RFS between the two arms in 567 patients (366/201) [10, 17]. Random effect model analysis revealed that OP was not independently associated with RFS of young patients with early-stage EC (HR 1.22, 95% CI 0.32–4.72, *P* = 0.648 Figure 3), with statistically significant heterogeneity detected between studies (*I*^2^ = 50.3%, *P* = 0.156). Funnel plot showed that there might be publication bias (Egger test, *P* = 0.024, Begg test, *P* = 0.296).

**Figure 4:**
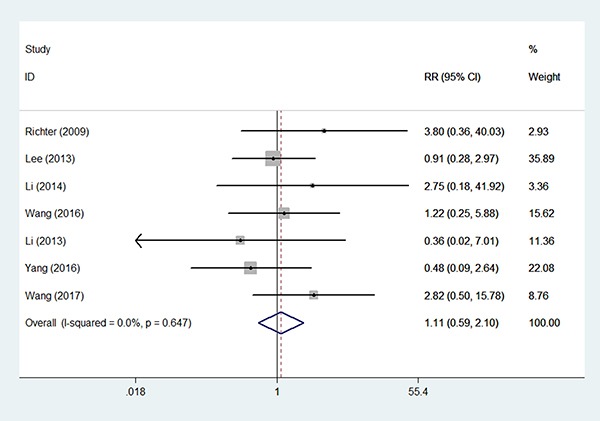
Forest plots illustrate the intervention results of the RRs for RFS

**Figure 5:**
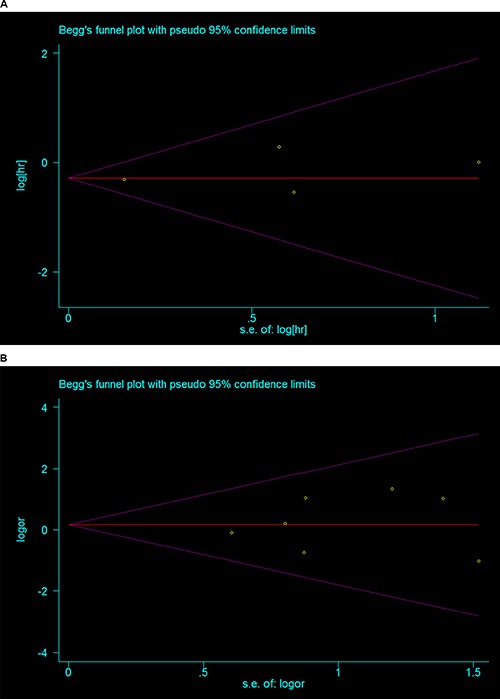
Begg's Funnel plot of the included studies (**A**) OS-HR (**B**) RFS-RR.

Only two studies reported data on HR of RFS, and these data were too sparse to be pooled. We extracted data to calculate a pooled RR and 95% CI. Similarly, for seven studies with 1024 patients enrolled (706/318) [10, 12–14, 16–18], fixed effect model analysis (*I*^2^ = 0.00%, *P* = 0.647) did not show any significant benefit in the BSO group over the OP group with respect to RFS (RR 1.11, 95% CI 0.59–2.10, *P* = 0.745 Figure 4, Supplementary Table 1), with no evidence of publication bias (Egger test, *P* = 0.633, Begg test, *P* = 1.000) (Figure 5B). There were 13 patients (4.09%) who experienced disease recurrence in the OP group and 23 patients (3.26%) in the BSO group. Considering three of seven studies enrolled some patients with a non-endometrioid histological type, we carried out subgroup analyses excluding these three studies, and we still found no significant effect of OP on RFS (RR 1.10, 95 CI 0.51–2.37, *P* = 0.811) with no statistically significant heterogeneity detected between studies (*I*^2^ = 0.0%, *P* = 0.457).

## DISCUSSION

Since no prospective study on this issue has been designed and a randomized controlled trial is unlikely in the near future owing to the anticipated difficulty in patient recruitment, the results of this study carry weight. This meta-analysis, which included over 10,000 cases from the USA, Korea, and China, demonstrates that OP significantly improves the OS (HR 0.75, 95% CI 0.57–0.99) and does not adversely impact RFS (RR 1.11, 95% CI 0.59–2.10) of young patients with early-stage EC. Although this study cannot conclusively refute or support the safety of OP with regard to risk of EC recurrence (RR), it is noteworthy that, in this low risk population, the absolute recurrence rate (4.09%) was low. Patients who receive surgery with ovarian conservation might take a 2.1 fold risk of recurrence, but in the long run they will benefit and have a higher OS probability.

Two previous systematic reviews have examined the survival benefit of ovarian conservation in endometrial carcinoma [41, 42]. Both had limitations. Neither limited the patient age or the histological type of tumor, and no significant impact of OP on survival was found. Both extracted data from articles published no later than April 2016, and new big data research published after that were not included. One of them extracted and pooled HRs that they estimated from Kaplan-Meier curves, which rendered the findings potentially inaccurate. Because the pooled HR of RFS was only calculated from 3 studies, we could say that small numbers of studies led the meta-analysis results to be improperly evaluated. The other study included some overlapping articles published from the same database, and this methodological error could lead to bias.

As the proportion of young women diagnosed with EC has been rising over time, it has become increasingly important to reach consensus on the practice of OP. Between 1983 and 2012, approximately 15% of patients diagnosed with stage I type I EC were under the age of 50 and were forced to make a difficult choice on whether or not to receive an oophorectomy [19]. The safety of ovarian conservation in young women with EC has been questioned based primarily on two theoretic concerns. First is the possibility that the ovary coexists with metastatic disease from EC or a synchronous primary tumor of the ovary. Synchronous endometrial and ovarian cancers have been reported to occur in 5%~25% of premenopausal women with EC, but typically present as enlarged masses or gross adnexal abnormalities that can be detected clinically or at time of surgery [6, 7, 24]. Lee et al. found that among the 206 patients without any evidence of intraoperative extrauterine disease, the coexisting ovarian malignancy rate was 0.97%, and zero for those under age of 45 [7]. Ken et al. also reported that ovarian involvement occurred in 5% of patients with clinical stage I EC, and microscopic ovarian involvement without grossly visible lesions only occurred in 0.8% of the patients [25].

The second concern stems from the possibility that continued estrogen production by the ovaries may stimulate residual endometrial tumor cells. Four retrospective studies have looked at the issue of estrogen replacement therapy (ERT) after surgical treatment of early-stage endometrial adenocarcinoma, and all of them show no significant increase in recurrences or deaths caused by EC in the ERT group [26–29]. A prospective randomized controlled trial of ERT by the Gynecologic Oncology Group (GOG 317), although ended early, found no increased risk of recurrence or death in the ERT group compared with the placebo group (RR 1.27, 80% CI 0.92–1.77), and the incidence of new malignancy was low [30].

It has been suggested that surgical menopause can cause adverse long-term effects in bone, heart, and neurologic health as well as quality of life. Additionally, it eliminates the fertility of young nulliparous women completely [5, 31, 32]. Several studies reported that early oophorectomy has a direct effect on all-cause mortality. A prospective, population-based cohort study found that women who underwent prophylactic bilateral oophorectomy prior to the age of 45 years had a 67% increase in mortality [32]. In a recent meta-analysis, the relative risk of cardiovascular disease in women who had undergone BSO was 2.62 [31]. Rosenberg et al. reported that the risk of myocardial infarction is increased more than seven-fold in those who undergo bilateral oophorectomy prior to the age of 35 years [33]. Younger patients with EC are characterized by obesity, nulliparity, chronic anovulation, and the presence of polycystic ovarian syndrome or metabolic syndrome, which place them at high risk of cardiovascular disease, hence, preservation of the ovaries may be protective. Koskas et al. found that, in observation of 489 EC patients, heart disease or diabetes-related deaths only occurred in patients who underwent oophorectomy [8]. Koji et al. even demonstrated OP is an independent predictor for decreased risk of death from cardiovascular disease among women aged younger than 50 years with stage I grade I endometrioid EC (HR 0.40, 95% CI 0.17–0.91) [19]. As assisted reproductive technology has developed, OP also protects fertility for young patients with EC. Wang et al. indicated that the OP group had a significantly higher quality of sexual life and better level of sex hormones [13]. Similarly, Yang et al. demonstrated that patients with OP had a better post-operative life according to Kupperman Score and FACT score [12].

Although current guidelines recommend oophorectomy for all women with EC, mounting data suggests that it may be time to reevaluate these recommendations and consider more individualized treatment, especially for early-stage young patients. In an observation study of Lee et al, seven of the 175 EC patients had documented recurrence, and all seven recurrences had risk factors, namely, non-endometrioid histology, deep myometrial invasion, cervical stromal invasion, and inadequate adjuvant treatment [34]. Yoshino et al. confirmed that OP surgery might be considered in EC of endometrioid histology with ≤ 50% myometrial depth invasion with no ovarian mass after taking into account family history [35]. Data from the SEER database which covers approximately 27.8% of the U.S. population found that, patients with stage I G1 tumors had a significantly longer OS with OP [19]. Accordingly, the indications for OP of EC patients include: patients under age of 50; early-stage carcinoma with no deep myometrial invasion; low-grade endometrial cancer of endometrioid histological subtype; no gross mass of ovary or ultra-uterine lesion in operation; no family history.

In regard to the limitations of this study, first, all the studies enrolled in the meta-analysis were retrospective data, and some did not have a sufficient follow-up period due to missing data in the medical records. Despite the recognized limitations of observational data, all of the studies in our analysis are high quality according to NOS, and it is unlikely that a randomized controlled trial of oophorectomy compared with ovarian conservation will ever be performed. Second, as in any observational study, a number of unmeasured confounders may have influenced the allocation of treatment. We lack data on family history, the presence of inherited genetic abnormalities such as Lynch syndrome, body mass index, the gross appearance of the ovaries at the choice to perform oophorectomy, and the status of adjuvant treatment. Third, the consequences of surgical menopause in young women, such as cardiovascular disease, osteoporosis, and quality of life, such as hot flushes and genital tract atrophy, were not evaluated. Fourth, publication bias might exist due to the small number of studies that were included for each outcome, although the assessment of publication bias showed no statistically significant asymmetry. Fifth, although we collected data from China to calculate in our study, these data were not population-based, which might lead to selection bias. Finally, some of the patients had ovaries saved incidentally because they were not diagnosed as having EC preoperatively, while others were diagnosed with EC preoperatively and were willing to conserve their ovaries with informed consent. OP is more like to be performed when a clinician encounters a favorable case and a younger patient. Thus, selection bias should have been controlled in order to compare the survival outcomes of the two groups.

In summary, this meta-analysis demonstrates that OP significantly improves the OS and does not significantly decrease RFS of young patients with early-stage EC. OP is a reasonable option in some young women with low risk EC after a thorough preoperative evaluation and an extensive intraoperative exploration. Further larger trials to evaluate the safety of OP for low risk EC patients (stage Ia, G1 endometrioid adenocarcinoma) with adequate survival data, cardiovascular disease, osteoporosis, and quality of life measured, especially from Asia, are warranted.

## MATERIALS AND METHODS

### Search strategy

This systematic review and meta-analysis is reported in accordance with the Preferred Reporting Items for Systematic Reviews and Meta-Analyses (PRISMA) Statement and was registered at International Prospective Register of Systematic Reviews (number CRD42017054306). We identified observational studies by searching Medline (from 1950), Embase (from 1980), Cochrane library (from 1996), the Chinese Biomedicine Literature Database (from 1978), the Chinese Scientific Journal Full-text Database (from 1989), the Chinese Journal Full-text Database (from 1994), and the Wanfang Database (from 1980). The search strategy included terms for endometrial cancer (endometrial cancer, endometrial carcinoma, endometrial neoplasm, endometrium cancer, endometrium carcinoma, and endometrium neoplasm), patient age (premenopausal, young, and reproductive age), tumor stage (early-stage, stage I, and stage II), and treatment (ovarian conservation and OP). The literature search was performed up to March 2017. The full electronic search strategy for Medline can be found in the appendix (Supplementary Appendix 1). We hand searched abstract books of conference proceedings between 2010 and 2016 to identify potentially eligible studies. The reference lists of all identified relevant studies were used to carry out a recursive search of the literature.

### Selection criteria

Retrieved articles had to meet the following inclusion criteria: (1) Patients who underwent hysterectomy and were diagnosed with Stage I or II endometrial carcinoma; (2) premenopausal or under the age of 50; (3) original articles comparing the OS and/or RFS between the group treated with ovarian preservation surgery (OP group) and the group that received BSO (BSO group); and (4) only studies published in English or Chinese were included. This age cutoff of 50 years was chosen based on mean age of spontaneous menopause in the North American and Chinese populations [36, 37].

The following were the exclusion criteria: (1) Uterine sarcomas and metastatic tumors to the uterus were excluded. (2) Patients with EC that coexisted with other malignant tumors were excluded. (3) Articles without full-text or articles without extractable data to calculate were excluded. (4) The study year, study centers, and study periods were investigated and overlapping articles were excluded.

Two authors (P.J. and Y.Z) independently examined the titles and abstracts of all articles, and excluded those going beyond the selection criteria. Any discrepancy in their opinions was discussed to reach an agreement.

### Data extraction

Two reviewers independently extracted data on to an Excel spreadsheet (Microsoft for Mac 2011) as survival outcomes (HR of OS and RFS, total number of death, or recurrence events). In addition we extracted the following clinical data for each trial: country, period of observation, patient age, tumor stage, the grade and histology of tumor, and duration of follow-up.

### Methodological quality assessment

No prospective study on this issue has been designed, thus only observational studies were included in the meta-analysis. To evaluate the quality of the studies, two reviewers (P.J. and Y.Z) assessed studies according to the NOS [38] for observational studies independently. The articles graded with more than 6 stars on the NOS were considered to be of high quality.

### Statistical analysis

The results of this study were expressed as a pooled HR and 95% CI. A value of *P* < 0.05 was considered to be statistically significant. For too few studies expressing RFS outcomes as HR, we also extracted data to calculate a pooled RR and 95% CI. Study-to-study variation was assessed using the Higgins I^2^ test, which measured the proportion of the total variation across the studies [39]. When significant heterogeneity(*P*-value < 0.1 or I^2^ > 25% [39]) was not observed between the studies in the meta-analysis, the fixed effects model was used, and when significant heterogeneity was observed, the random effects model was used. We planned to do sensitivity analyses *a priori* according to patient age and histology type.

We used STATA 12.0 (Stata Corp, College Station, TX) to generate forest plots of pooled HR, RR, and risk differences for outcomes with 95% CI. The Egger and Begg tests were used to assess funnel plots for evidence of publication bias [40].

## SUPPLEMENTARY MATERIALS TABLES


