# Supplementary material for: Ovarian preservation improves overall survival in young patients with early-stage endometrial cancer
Source: Oncotarget. 2017 Jun 7;8(35):59940–9. doi: 10.18632/oncotarget.18404 (PMC5601791; doi:10.18632/oncotarget.18404)
Supplement: Supplementary file 1 [file oncotarget-08-59940-s001.pdf]

Ovarian preservation improves overall survival in young patients with early-stage endometrial cancer

Supplementary Materials

Supplementary Appendix 1: search strategy for pubmed

Search (((((prognosis [MeSH:noexp] OR diagnosed [Title/Abstract] OR cohort* [Title/Abstract] OR cohort effect [MeSH Term] OR cohort studies [MeSH:noexp] OR predictor* [Title/Abstract] OR death[Title/Abstract] OR “models, statistical”[MeSH Term]))) AND ((((((((premenopausal [Title/Abstract]) OR young [Title/Abstract]) OR reproductive age [Title/Abstract]) OR early stage[Title/Abstract]) OR stage I [Title/Abstract]) OR stage II [Title/Abstract]) OR reproductive-age [Title/Abstract]) OR early-age [Title/Abstract])) AND ((ovarian conservation [Title/Abstract]) OR ovarian preservation [Title/Abstract])) AND ((((((((((((((((((endometrial neoplasm[Title/Abstract]) OR neoplasm, endometrial [Title/Abstract]) OR neoplasms, endometrial [Title/Abstract]) OR endometrial carcinoma [Title/Abstract]) OR carcinoma, endometrial [Title/Abstract]) OR carcinomas, endometrial [Title/Abstract]) OR endometrial carcinomas [Title/Abstract]) OR endometrial cancer [Title/Abstract]) OR cancer endometrial [Title/Abstract]) OR cancers, endometrial [Title/Abstract]) OR endometrial cancers [Title/Abstract]) OR endometrium cancer [Title/Abstract]) OR cancer, endometrium [Title/Abstract]) OR cancer of the endometrium [Title/Abstract]) OR carcinoma of endometrium [Title/Abstract]) OR endometrium carcinoma [Title/Abstract]) OR endometrium carcinomas [Title/Abstract]) OR cancer of endometrium [Title/Abstract])) OR “Endometrial Neoplasms”[Mesh]).

Supplementary Table 1: Recurrence of patients with OP in EC

study	year	NO. Patients			
		OP group		BSO group	
		Recurrence	Total	Recurrence	Total
**Wang**	2017	3	25	2	47
**Richter**	2009	1	20	2	153
**Lee**	2013	4	176	8	319
**Li**	2014	1	20	1	55
**Wang**	2016	2	25	5	76
**Li**	2013	0	17	2	31
**Yang**	2016	2	35	3	25
